# Factors Associated with Adolescents’ Internet Use Duration by Suicidal Ideation

**DOI:** 10.3390/ijerph17020433

**Published:** 2020-01-09

**Authors:** Myoungjin Kwon, Sun Ae Kim, Wi-Young So

**Affiliations:** 1 Department of Nursing, Daejeon University, Daejeon 34520, Korea; mjkwon@dju.kr; 2 Department of Nursing, Korea National University of Transportation, Chungbuk 27909, Korea; 3 Sports and Health Care Major, College of Humanities and Arts, Korea National University of Transportation, Chungju-si 27469, Korea

**Keywords:** adolescents, Internet use, Korea Youth Risk Behavior Web-Based Survey, suicidal ideation

## Abstract

*Purpose*: This study aimed to identify the factors associated with Internet use duration by distinguishing between individuals with suicidal ideation and those without. *Methods*: Data were obtained from the 14th Korea Youth Risk Behavior Web-Based Survey (2018), which is a nationwide survey. Respondents aged 12–18 years (N = 60,040) who answered “yes” or “no” to the question about having suicidal ideation were included in the analysis. Study variables were general characteristics, physical and psychological factors, and Internet use duration. A complex sample logistic regression was performed to determine the influential factors. *Results*: Significant factors associated with weekend Internet use among those with suicidal ideation were sex, economic status, residence type, physical activity, sedentary duration, recovery after sleep deprivation, and stress, with an explanatory power of 20.0% (*p* < 0.001). Significant factors associated with weekday Internet use among those with suicidal ideation were sex, frequency of eating breakfast per week, sedentary duration, and weight control efforts, with an explanatory power of 15.9% (*p* < 0.001). Significant factors associated with weekend Internet use among those with no suicidal ideation were breakfast frequency and frequency of alcohol consumption per month, with an explanatory power of 10.9% (*p* < 0.001). Significant factors associated with weekday Internet use among those with no suicidal ideation were frequency of eating breakfast per week, frequency of eating fast food per week, sedentary duration, and suicide attempt, with an explanatory power of 13.6% (*p* < 0.001). *Conclusions*: The findings indicated significant differences in Internet use duration between adolescents with suicidal ideation and those without, suggesting the need for customized prevention programs focusing on adolescents’ psychological state.

## 1. Introduction

Adolescence is a vital period of human development, because it is the period between childhood and adulthood and involves substantial physical growth and brain development [1]. However, it is also marked by emotional and psychological instability, and the prevalence of depression and anxiety disorders in adolescence is very high [2]. Suicide is the main cause of adolescent death in Korea and the second most common cause of adolescent death in the United States [3,4]. Moreover, death rates by suicide are rising exponentially [5].

Suicidal ideation is defined as having serious thoughts about suicide; thus, it is considered an important risk factor for suicidal attempts [6,7]. Moreover, it is a predictor of other negative outcomes such as depression, poor mental health, and drug use [8]. The identified factors associated with suicide have a greater influence on adolescents than on adults [9]. Excessive Internet use such as Internet addiction is one of the factors related to suicidal ideation and attempts among adolescents [10], although the effects of the Internet are still controversial. The Internet has become an indispensable tool, and 90% of the population use the Internet in Korea, Australia, and Canada [11]. Despite multiple advantages of the Internet, excessive Internet use can cause Internet addiction, which can lead to other psychological problems [12].

The most important issues with respect to Internet use are its management and regulation. According to a meta-analysis of studies on Internet addiction conducted in 31 countries, the overall Internet addiction prevalence rate was around 6.0%, which is very high compared to the 0.2–2.1% prevalence rate for pathological gaming addiction [13]. Moreover, in countries reporting high levels of Internet addiction, overall life satisfaction was low, pollution was severe, and the economic conditions were poor [13]. Furthermore, mental illnesses such as depression and anxiety have been frequently reported among adolescents who overuse the Internet [14,15]. In addition, adolescents who are at an increased risk of Internet addiction are at higher risk for mental health problems [16].

The Diagnostic and Statistical Manual of Mental Disorder, 5th edition (DSM-5) recognizes only “Internet Gaming Disorder,” which was added as a new disorder in this revision [17]. However, Internet overuse or addiction has not yet been identified as a disease, and accurate standards for its diagnosis are not yet determined. Furthermore, to date, no studies have reported a clear relationship between Internet use duration and suicidal ideation among adolescents. Is there a difference in Internet use duration between adolescents who have suicidal ideation and those who do not? If excessive Internet use leads to Internet addiction, it might affect one’s psychological and social characteristics as well. Therefore, this study was conducted to identify the factors associated with Internet use duration for those who have suicidal ideation and those who do not. The findings are expected to provide basic data for research on Internet usage time management, policy development, and development of customized intervention programs for adolescents based on suicidal ideation.

## 2. Method

### 2.1. Participants

This secondary analysis used data from the 14th Korea Youth Risk Behavior Web-Based Survey in 2018. The survey was conducted by the Korea Centers for Disease Control and Prevention to examine the health behaviors of middle- and high-school students. All data were anonymous and self-reported, and one computer was randomly assigned to each respondent in a computer room of an Internet-enabled school.

Respondents aged 12–18 years (N = 60,040) who answered “yes” or “no” to the question of “Have you ever seriously thought about suicide for a two-week period in the last 12 months?” were included in the analysis. All statistical analyses were conducted using sample weights assigned to the participants. The Korea Youth Risk Behavior Web-Based Survey is approved by government-mandated statistics based on the Bioethics Law. All participants voluntarily participated and provided written informed consent. As this study involved secondary data analysis, individual ethical approval was not required.

### 2.2. Measures

The variables examined in this study were general characteristics and physical and psychological characteristics.

#### 2.2.1. General Characteristics

General characteristics included age, sex, academic grades, economic status, frequency of eating breakfast/week, frequency of eating fast food/week, residence type, Internet use duration (min/day) on weekends, and Internet use duration (min/day) on weekdays.

Age was categorized into 12–15 years and 15–18 years. Academic achievement and economic status each were classified as low, middle, and high. Frequency of eating breakfast and fast food each were classified as 0, 1–3, and 4 times or more per week. Residence types included living with family; in a childcare facility; and in a relative’s house, dormitory/rental, or boarding.

#### 2.2.2. Physical Characteristics

Physical factors included physical activity per week, sedentary duration (min per day), body mass index (BMI; kg/m^2^), weight control efforts, recovery after sleep deprivation, frequency of alcohol consumption, amount of alcohol consumption (number of glasses of *soju*, Korean distilled spirit), smoking experience, number of cigarettes per day, sexual experience, and experience of recreational drug use.

Physical activity was classified as frequency of engaging in physical activities for over 60 minutes: 0, 1–3, and 4 times or more per week. Sedentary duration was classified as less than 90 minutes, 91–180 minutes, and 181 minutes or more per day. BMI was categorized as less than 18.5 kg/m^2^, 18.5–24.9 kg/m^2^, and 25 kg/m^2^ or more. Weight control effort was divided into no effort, effort to reduce weight, effort to increase weight, and effort to maintain weight. Recovery after sleep deprivation was categorized into sufficient, normal, and not sufficient. Responses about alcohol consumption were divided into “yes” and “no.” Frequency of alcohol consumption was divided into less than 2 times, 3–9 times, and 10 times or more per month. Amount of alcohol consumption/number of glasses of *soju* was divided into less than 2 glasses of *soju*, 3–6 glasses of *soju*, and more than one bottle of *soju* at once. Responses about smoking experience were divided into “yes” and “no.” Number of cigarettes per day was divided into less than 1 cigarette, 2–9 cigarettes, and 10 cigarettes or more per day. Responses about sexual experience were divided into “yes” and “no.” Drug use experience was divided into “yes” or “no.”

#### 2.2.3. Psychological Characteristics

Psychological factors included stress (“high stress,” “moderate stress,” “little stress,” “almost no stress,” and “no stress”), feelings of sadness for two weeks (“yes” or “no”), subjective health status (healthy, moderate, not healthy), subjective body perception (thin, normal, overweight), suicidal ideation (“yes” or “no”), and suicidal attempt (“yes” or “no”).

### 2.3. Statistical Analysis

Using the IBM SPSS 25.0 program (IBM Corp., Armonk, NY, USA), weights were assigned to generate a composite sample plan file, which was then analyzed. The significance level was set at *p* < 0.05. General, physical, and psychological characteristics of the participants were analyzed using crosstabs and chi-square tests. A complex sample logistic regression was performed to examine the factors associated with suicidal ideation by weekend and weekday Internet use.

## 3. Results

### 3.1. General Characteristics of Participants

There were significant differences between the two groups with respect to sex, academic performance, economic level, breakfast per week, fast food intake per week, residence type, and weekend and weekday Internet use time (*p* < 0.001). Compared to adolescents who did not have suicidal ideation, those with suicidal ideation had a higher number of female students (*p* < 0.001), low academic grades, and low economic status (*p* < 0.001). Furthermore, adolescents who had suicidal ideation had a greater frequency of skipping breakfast and consumed fast food more than four times a week. They were also more likely not to live with their families and spent more time on the Internet on both weekdays and weekends (Table 1).

### 3.2. Physical and Psychological Characteristics of Participants

Significant differences were found between the two suicidal ideation groups with respect to the frequency of engaging in physical activity for more than 60 minutes per week, sedentary duration, weight control efforts, recovery after sleep deprivation in the past seven days, frequency of alcohol consumption, amount of alcohol consumption, smoking experience, frequency of smoking, sexual experience, and experience of recreational drug use (*p* < 0.05). Adolescents with suicidal ideation performed physical activity less frequently, spent more sedentary duration, and more often tried to lose weight. In addition, they were more likely to not recover from sleep deprivation, consume 10 drinks or more a month, and smoke 10 cigarettes or more a day. Moreover, a greater proportion of adolescents with suicidal ideation reported sexual activities and drug use experience (Table 2). Regarding psychological characteristics of both groups, there were significant differences with respect to stress, feelings of sadness in the last two weeks, subjective health status, subjective body perception, suicidal ideation, and suicidal attempt (*p* < 0.001). Adolescents with suicidal ideation reported more stress (*p* < 0.001) and more sadness (*p* < 0.001). Adolescents with suicidal ideation were more likely to perceive their subjective health status to be worse (*p* < 0.001) and their subjective body perception to be more obese (*p* < 0.001). In addition, suicide ideations and suicide attempts were more frequent in groups with suicidal ideation (*p* < 0.001) (Table 2).

### 3.3. Factors Associated with Internet Use Duration by Suicidal Ideation

Significant factors associated with weekend Internet use time among those with suicidal ideation were sex, economic status, residence type, physical activity, sedentary duration, recovery after sleep deprivation, and stress, with an explanatory power of 20.0% (*p* < 0.001). Internet use duration was longer among female adolescents (*p* = 0.009) and adolescents with middle- (*p* = 0.012) or high economic status (*p* = 0.028). Moreover, adolescents with suicidal ideation did not live with family members (*p* = 0.019), had more sedentary duration (*p* < 0.001), had enough recovery after sleep deprivation (*p* = 0.013), and almost never had stress compared to adolescents who reported no stress at all (*p* = 0.028). Significant factors for weekday Internet use time among those with suicidal ideation were sex, frequency of eating breakfast per week, sedentary duration per day, and weight control efforts, with an explanatory power of 15.9% (*p* < 0.001). Internet use duration was longer among male adolescents (*p* = 0.010), and adolescents who consumed breakfast less frequently (*p* = 0.037). Those who tried to gain weight spent less time using the Internet (*p* = 0.029) (Table 3).

Significant factors for weekend Internet use time among those without suicidal ideation were frequency of eating breakfast, sedentary duration per day, and alcohol consumption per month, with an explanatory power of 10.9% (*p* < 0.001). Compared to those who ate breakfast 4 times or more a week, those who do not eat breakfast at all reported longer Internet use duration (*p* < 0.001). Furthermore, the shorter the sedentary duration, the shorter was the Internet use duration (*p* < 0.001). Those who drank less than two times and who drank between three and nine times spent less time on Internet use than those who drank more than 10 times a month (≤2/month *p* = 0.029, 3–9/month *p* = 0.024). Significant factors for weekday Internet use duration among those with no suicidal ideation were frequency of eating breakfast per week, frequency of eating fast food per week, sedentary duration, and suicide attempt, with an explanatory power of 13.6% (*p* < 0.001). Participants who did not consume breakfast at all reported longer Internet duration than those who consumed more than four breakfast meals per week (*p* = 0.016). Participants who did not eat fast food at all (*p* = 0.038) and who ate fast food 1–3 times per week (*p* = 0.024) spent less time on the Internet compared to those who consumed fast food four times or more per week. The shorter the sedentary duration (*p* < 0.001), the shorter was the Internet use duration, and participants who had not attempted suicide had a longer Internet use duration (*p* = 0.035) (Table 3).

## 4. Discussion

The present study identified general characteristics and physical and psychological factors associated with Internet use among adolescents by the presence or absence of suicidal ideation. The findings showed that alcohol consumption and drug use were significantly more frequent among adolescents with suicidal ideation than adolescents without suicidal ideation. Furthermore, stress and depression were higher among adolescents with suicidal ideation. Adolescents with suicidal ideation reported having more frequent suicidal perceptions and attempts. Although most participants in both groups reported that they did not perception to attempt suicide and had not attempted suicide, those with suicidal ideation were more likely to perception and attempt suicide. There was a statistically significant difference in the perception and attempt of suicide depending on the suicidal ideation.

Previous studies, with suicides differed by sex, have indicated that boys are more likely to commit suicide in adolescence and girls are more likely to commit suicide after adolescence. In addition, suicidal ideation, previous suicide attempts, intentions and motivations, and mental disorders have been identified as risk factors for adolescent suicide [18]. Furthermore, a study by Mars et al. found that adolescents with suicidal ideation and suicide attempts were more likely to engage in self-harm and reported more psychiatric disorders, such as depression, anxiety disorder, and behavioral disorder [19]. Although a previous study found a link between actual suicide attempts and suicidal thoughts [20], there is still insufficient clear evidence for this relationship.

A recent study has shown that “suicide capability”—the perception that one can actually attempt suicide—is a factor that operates between suicidal ideation and actual suicide attempts [20]. However, the research on suicide capability is very limited and more research is needed. Thus, it is clear that there is a connection between suicidal ideation and actual suicides, and more research on the factors associated with them should be conducted. Understanding this relationship would help to address the factors associated with suicidal attempts and thereby prevent actual suicides, especially considering that adolescents might be able to address some of these factors by themselves.

The present study found significant differences in the factors associated with Internet use duration during weekdays and weekends by suicidal ideation. These findings suggest new directions for intervention development or preventive strategies. The average weekend Internet duration was 228 minutes (3.8 hours) among those without suicidal ideation and 266 minutes (4.4 hours) among those with suicidal ideation. These results were statistically significant, but the difference between the actual 3.8 hours and 4.4 hours may not be recognized as an obvious difference. Therefore, these results should be interpreted carefully. However, the American Academy of Pediatrics recommends using screen media less than two hours a day [21]. In particular, this study found that adolescents with suicidal ideation reported longer Internet use duration, which exceeded the suggested usage time as well as the average reported in previous studies. These results indicate that adolescents spend considerable time on the Internet during weekend breaks. Furthermore, excessive smartphone use is associated with sleep disorders, depression, and chronic stress [22]. In addition, adolescents are susceptible to following the harmful behaviors of their peers, which can aggravate problem behaviors [23].

This study has several limitations. First, secondary data were analyzed, and thus, it was impossible to change the measurement variables. Therefore, research including additional qualitative data on Internet use is needed. Second, there was also a substantial difference between the proportions of adolescents with suicidal ideation and those without suicidal ideation, although this is expected. Therefore, evidence-based results from interventional studies with similar proportions of adolescents with and without suicidal ideation are needed. Despite these limitations, the results of this study are highly representative of all Korean adolescents, because the survey was conducted at the national level, and the entire process was designed to reflect representativeness. In addition, detailed basic data for the development of guidelines for the appropriate use of the Internet through future research are needed. Furthermore, by establishing a model that can predict suicidal ideation or actual suicides based on the collected characteristics and patterns of the participants, studies that identify predictable paths will not only help adolescents with suicidal ideation but also effectively prevent suicides.

## 5. Conclusions

This study showed a difference in the Internet use duration between adolescents who engage in suicidal ideation and those who do not. The results also confirm the need for prevention programs that focus on adolescents, particularly those vulnerable to the risk of psychological problems, such as suicidal ideation. This study provides a better understanding of the associations between suicidal ideations and Internet use and could be used as the basis to develop programs for preventing Internet overuse. Finally, although the difference in the amount of time spent on the Internet may not be considered as decisive factor of suicidal attempts, it is beneficial to help adolescents engage in meaningful activities other than using the Internet.

## Figures and Tables

**Table 1:** General characteristics of the participants by presence or absence of suicidal ideation.

Characteristics		n (Weighted %)	No (n = 52,064)	Yes (n = 7976)	x^2^/*t* (*p*)
			n (Weighted %) or Complex Sample Mean		
Age (years)	12–15	34,657 (54.4)	30,006 (54.2)	4651 (55.6)	5.21 (0.094)
	16–18	25,077 (45.6)	21,848 (45.8)	3229 (44.4)	
Sex	Male	30,463 (52.1)	27,639 (54.3)	2824 (37.4)	801.58 (<0.001)
	Female	29,577 (47.9)	24,425 (45.7)	5152 (62.6)	
Academic grades	High	23,420 (38.8)	20,708 (39.5)	2712 (33.9)	261.48 (<0.001)
	Middle	17,526 (29.4)	15,434 (29.8)	2092 (26.3)	
	Low	19,094 (31.9)	15,922 (30.7)	3172 (39.8)	
Economic status	High	24,207 (40.8)	21,429 (41.6)	2778 (35.8)	567.50 (<0.001)
	Middle	27,808 (46.0)	24,362 (46.5)	3446 (42.7)	
	Low	8025 (13.2)	6273 (11.9)	1752 (21.5)	
Frequency of breakfast/week	0	11,128 (18.7)	9461 (18.3)	1667 (21.1)	96.88 (<0.001)
	1–3	13,739 (22.8)	11,682 (22.4)	2057 (25.4)	
	≥4	35,173 (58.5)	30,921 (59.3)	4252 (53.5)	
Frequency of eating fast food/week	0	11,676 (19.0)	10,147 (19.0)	1529 (18.7)	117.17 (<0.001)
	1-3	45,878 (76.7)	39,946 (77.0)	5932 (74.6)	
	≥4	2486 (4.3)	1971 (4.0)	515 (6.8)	
Residence type	With family	56,654 (95.5)	49,248 (95.7)	7406 (94.2)	109.23 (<0.001)
	Childcare facility	2436 (3.3)	2119 (3.3)	317 (3.4)	
	Boarding home, relative’s home, dorm, home of relative	711 (1.2)	525 (1.0)	186 (2.3)	
Internet use duration (min/day on weekends)			228.57	266.59	−14.77 (<0.001)
Internet use duration (min/day on weekdays)			149.80	179.03	−13.47 (<0.001)

**Table 2:** Physical and psychological characteristics of the participants by presence or absence of suicidal ideation.

Characteristics			n (Weighted %)	No (n = 52,064)	Yes (n = 7976)	x^2^ (*p*)
				n (Weighted %)		
Physical factors	Physical activity (/week)	0	21,562 (36.3)	18,550 (36.0)	3012 (38.1)	26.96 (<0.001)
		1–3	25,976 (43.3)	22,489 (43.3)	3487 (43.5)	
		≥4	12,502 (20.4)	11,025 (20.7)	1477 (18.4)	
	Sedentary duration (min)	≤90	16,068 (28.0)	14,152 (28.4)	1916 (25.3)	109.83 (<0.001)
		91–180	23,709 (41.8)	20,769 (42.2)	2940 (39.3)	
		≥181	17,561 (30.2)	14,869 (29.4)	2692 (35.4)	
	Body mass index (kg/m^2^)	<18.5	11,910 (20.6)	10,429 (20.6)	1481 (20.1)	6.55 (0.178)
		18.5–22.9	29,616 (52.1)	25,800 (52.2)	3816 (51.3)	
		23–24.9	6912 (12.2)	6000 (12.2)	912 (12.7)	
		≥25	8556 (15.2)	7392 (15.0)	1164 (16.0)	
	Weight control effort	No effort	28,091 (47.3)	24,938 (48.4)	3153 (40.4)	263.74 (<0.001)
		Lose weight	20,468 (33.6)	17,077 (32.4)	3391 (41.5)	
		Gain weight	4164 (7.1)	3678 (7.2)	486 (6.4)	
		Maintain weight	7317 (12.1)	6371 (12.1)	946 (11.7)	
	Recovery after sleep deprivation	Enough	14,238 (23.0)	13,224 (24.7)	1014 (12.4)	1186.60 (<0.001)
		Moderate	20,098 (33.3)	17,907 (34.3)	2191 (27.1)	
		Not enough	25,704 (43.7)	20,933 (41.1)	4771 (60.5)	
	Alcohol consumption	Yes	24,697 (42.3)	20,513 (40.6)	4184 (52.4)	46.28 (<0.001)
		No	35,343 (57.7)	31,551 (59.4)	3792 (47.6)	
	Frequency of alcohol consumption	≤2/month	20,508 (82.5)	17,279 (83.7)	3229 (76.4)	142.55 (<0.001)
		3–9/month	2998 (12.5)	2364 (11.9)	634 (15.2)	
		≥10/month	1191 (5.1)	870 (4.4)	321 (8.3)	
	Amount of alcohol consumption/number of glasses of *soju*	≤2	3768 (38.1)	3042 (38.7)	726 (35.9)	7.34 (0.058)
		3–6	2422 (25.2)	1899 (24.7)	523 (27.3)	
		≥7	3477 (36.7)	2764 (36.6)	713 (36.8)	
	Smoking experience	Yes	8540 (14.9)	6921 (14.0)	1619 (20.7)	224.45 (<0.001)
		No	51,500 (85.1)	45,143 (86.0)	6357 (79.3)	
	Frequency of smoking	≤1/day	1006 (26.3)	781 (26.2)	225 (26.5)	13.62 (0.003)
		2–9/day	1941 (52.4)	1546 (53.7)	395 (47.7)	
		≥10/day	775 (21.3)	575 (20.1)	200 (25.8)	
	Sexual Experience	Yes	3209 (5.7)	2469 (5.1)	740 (9.9)	257.73 (<0.001)
		No	56,831 (94.3)	49,595 (94.9)	7236 (90.1)	
	Experience of recreational drug use	Yes	260 (41.4)	135 (34.9)	125 (51.6)	16.35 (0.001)
		No	368 (58.6)	251 (65.1)	117 (48.4)	
Psychological factors	Stress	High stress	6925 (11.6)	3924 (7.6)	3001 (37.7)	7462.35 (<0.001)
		Moderate	17,387 (28.8)	14,022 (26.9)	3365 (41.6)	
		A little stress	24,638 (41.3)	23,294 (44.9)	1344 (17.3)	
		Almost no stress	9023 (14.9)	8833 (16.8)	190 (2.5)	
		No stress	2067 (3.4)	1991 (3.8)	76 (0.9)	
	Feelings of sadness	Yes	16,208 (27.1)	10,040 (19.3)	6168 (77.6)	1053.51 (<0.001)
		No	43,832 (72.9)	32,024 (80.7)	1808 (22.4)	
	Subjective health status	Healthy	43,300 (71.6)	39,218 (74.8)	4082 (51.1)	2157.66 (<0.001)
		Moderate	12,825 (21.6)	10,303 (20.1)	2522 (31.4)	
		Not healthy	3915 (6.7)	2543 (5.1)	1372 (17.5)	
	Subjective body perception	Thin	15,000 (25.3)	13,197 (25.6)	1803 (23.1)	229.21 (<0.001)
		Normal	21,848 (36.2)	19,371 (37.1)	2477 (30.8)	
		Overweight	23,192 (38.5)	19,496 (37.3)	3696 (46.1)	
	Suicide perception	Yes	2631 (4.4)	250 (0.5)	2381 (29.9)	8753.35 (<0.001)
		No	57,409 (95.6)	51,814 (99.5)	5595 (70.1)	
	Suicide attempt	Yes	1873 (3.1)	197 (0.4)	1676 (20.7)	5783.01 (<0.001)
		No	58,167 (96.9)	51,867 (99.6)	6300 (79.3)	

**Table 3:** Factors associated with Internet use duration on weekdays and weekends.

Model				Beta	Standard Error	*t*	*p*
Yes	Weekend	Sex	Male	−50.111	19.06	−2.62	0.009
			Female	1.000			
		Economic status	High	46.665	21.20	2.20	0.028
			Middle	55.781	22.09	2.52	0.012
			Low	1.000			
		Residence type	With family	−131.865	55.83	−2.36	0.019
			Day care center	−131.596	77.24	−1.70	0.089
			Boarding home, relative’s home, dorm, home of relative	1.000			
		Physical activity/week	0	−7.535	24.456	−0.30	0.758
			1–3	−48.005	22.027	−2.17	0.030
			≥4	1.000			
		Sedentary duration (min/day)	≤90	−104.910	25.284	−4.14	<0.001
			91–180	−94.162	20.628	−4.56	<0.001
			≥181	1.000			
		Recovery after sleep deprivation	Enough	100.849	40.38	2.49	0.013
			Moderate	10.069	19.91	0.50	0.613
			Not enough	1.000			
		Stress	High stress	73.084	67.91	1.07	0.282
			Moderate	68.790	68.61	1.01	0.317
			A little stress	57.337	71.45	0.80	0.423
			Almost no stress	218.655	99.33	2.20	0.028
			No stress	1.000			
	R^2^ = 0.200, F = 4.15, *p* < 0.001						
	Weekday	Sex	Male	−41.473	16.123	−2.57	0.010
			Female	1.000			
		Frequency of breakfast/week	0	−38.074	18.19	−2.09	0.037
			1–3	−9.056	17.59	−0.51	0.607
			4≤	1.000			
		Sedentary duration (min/day)	≤90	−76.769	21.80	−3.52	<0.001
			91–180	−81.363	16.03	−5.07	<0.001
			≥181	1.000			
		Weight control effort	No effort	−11.401	29.23	−0.39	0.697
			Weight loss	−26.641	30.35	−0.87	0.381
			Weight gain	−67.032	30.59	−2.19	0.029
			Keep weight	1.000			
	R^2^ = 0.159, F = 3.57, *p* < 0.001						
No	Weekend	Frequency of breakfast/week	0	42.567	11.52	3.69	<0.001
			1–3	6.709	10.08	0.66	0.506
			≥4	1.000			
		Sedentary duration (min/day)	≤90	−95.650	10.89	−8.77	<0.001
			91–180	−73.34	10.34	−7.09	<0.001
			≥181	1.000			
		Frequency of alcohol consumption	≤2/month	−30.028	13.67	−2.19	0.029
			3–9/month	−33.484	14.78	−2.26	0.024
			≥10/month	1.000			
	R^2^ = 0.109, F = 5.32, *p* < 0.001						
	Weekday	Frequency of breakfast/week	0	26.993	11.12	2.42	0.016
			1–3	−5.516	9.49	−0.58	0.562
			≥4	1.000			
		Frequency of eating fast food/week	0	−44.993	21.62	−2.07	0.038
			1–3	−43.754	19.26	−2.27	0.024
			≥4	1.000			
		Sedentary duration (min/day)	≤90	−96.562	10.46	−9.22	<0.001
			91–180	−79.917	7.93	−10.07	<0.001
			≥181	1.000			
		Suicide attempt	Yes	1.000			
			No	61.864	29.22	2.11	0.035
	R^2^ = 0.136, F = 6.11, *p* < 0.001						
